# Effect of Mannan-rich fraction supplementation on commercial broiler *intestinum tenue* and cecum microbiota

**DOI:** 10.1186/s42523-022-00208-6

**Published:** 2022-12-19

**Authors:** Robert J. Leigh, Aoife Corrigan, Richard A. Murphy, Fiona Walsh

**Affiliations:** 1 Antimicrobial Resistance and Microbiome Research Group, Department of Biology, Maynooth University, Co. Kildare, Ireland; 2 Alltech Inc. (Alltech European Bioscience Centre), Summerhill Road, Sarney, Dunboyne, Co. Meath, Ireland

## Abstract

**Background:**

The broiler gastrointestinal microbiome is a potent flock performance modulator yet may also serve as a reservoir for pathogen entry into the food chain. The goal of this project was to characterise the effect of mannan rich fraction (MRF) supplementation on microbiome diversity and composition of the *intestinum tenue* and cecum of commercial broilers. This study also aimed to address some of the intrinsic biases that exist in microbiome studies which arise due to the extensive disparity in 16S rRNA gene copy numbers between bacterial species and due to large intersample variation.

**Results:**

We observed a divergent yet rich microbiome structure between different anatomical sites and observed the explicit effect MRF supplementation had on community structure, diversity, and pathogen modulation. Birds supplemented with MRF displayed significantly higher species richness in the cecum and significantly different bacterial community composition in each gastrointestinal (GI) tract section. Supplemented birds had lower levels of the zoonotic pathogens *Escherichia coli* and *Clostridioides difficile* across all three *intestinum tenue* sites highlighting the potential of MRF supplementation in maintaining food chain integrity. Higher levels of probiotic genera (*eg. Lactobacillus* and *Blautia*) were also noted in the MRF supplemented birds. Following MRF supplementation, the cecum displayed higher relative abundances of both short chain fatty acid (SFCA) synthesising bacteria and SCFA concentrations.

**Conclusions:**

Mannan rich fraction addition has been observed to reduce the bioburden of pathogens in broilers and to promote greater intestinal tract microbial biodiversity. This study is the first, to our knowledge, to investigate the effect of mannan-rich fraction supplementation on the microbiome associated with different GI tract anatomical geographies. In addition to this novelty, this study also exploited machine learning and biostatistical techniques to correct the intrinsic biases associated with microbiome community studies to enable a more robust understanding of community structure.

**Supplementary Information:**

The online version contains supplementary material available at 10.1186/s42523-022-00208-6.

## Introduction

In recent years, the health impact of intestinal and cecal microbiome composition has become a prominent research focus in poultry science [29], 42]. Understanding and modulating the intrinsic and extrinsic interplay between differential microbial populations and their host environment has led to improved animal health and greater profitability in agricultural endeavours [23]. At present, broiler chickens (*Gallus gallus* subsp. *domesticus*; “broilers”) constitute the most consumed meat worldwide, with an approximate 100 million tons of poultry meat produced annually [67]. Due to their economic importance, high nutritive value, and accessibility of their meat, broilers have been extensively subjected to, and immensely benefitted from, intestinal microbiome composition and modulation analyses [11–13, 74]. The combined efforts of such research endeavours have reduced chick mortality, increased growth rates, and reduced the microbial load of major poultry and human pathogens [17, 52, 93]. Efforts of particular importance (and success) involve modulating microbiome composition using feed supplements [11].

The holobiont theory suggests that the health, metabolic prowess, and overall success and survivability of an organism is largely influenced by the composition, diversity, and complexity of their associated microbiomes [80]. Most previous studies of the chicken gut microbiome have focused on the ceca due to their dense bacterial populations which aid in digestion of otherwise indigestible residues remaining in chyme, bioconverting them to digestible metabolites for host absorption (eg. digestion of cellulose to glucose; [50, 69, 78, 84]). Many studies have found that differing microbiome compositions are strongly correlated with disease states across Metazoan lineages [22, 48, 78], and their modulation (via nutrient supplementation or transplantation) has resulted in profound improvements in human and animal health [11, 29, 52].

Due to increases in antimicrobial and metal or biocide resistance arising from their systematic use and misuse as livestock growth promoters and over-prescription in human medicine, alternative growth promotion techniques and supplements are being explored without using clinically relevant compounds [96]. One of the most promising poultry feed supplements are prebiotics containing mannan, such as mannan rich fraction (MRF) derived from *Saccharomyces cerevisiae* cell wall residues [8, 41]. These compounds display particular efficacy in binding to type-1 fimbriae in Gram-negative bacterial pathogens, specifically Enterobacteriaceae [28]. Reduction of such populations allows mutually symbiotic and commensal microbiota such as *Lactobacillus* to flourish [10, 29, 74, 93].

While 16S microbiome studies are highly informative, there may be some bias and lost significance due to the disparate number of 16S rRNA genes between species [95]. As these differences can be quite pronounced, we constructed a large 16S rRNA dataset from publicly available bacterial genomes and devised a simple weighting system, where each read from each taxon was divided by the number of 16S rRNA genes available in each taxon. The purpose of this procedure was to reduce bias caused by the widely uneven 16S rRNA gene counts commonly observed across Domain Bacteria.

Avian gut microbiome reports display considerable animal-to-animal variation which has the potential to incorrectly bias post hoc statistical comparisons [30, 99]. To counter this problem, we employed isolation forests (a common machine learning technique) and median imputation to each sample to remove and replace any outliers to decipher any previously unseen underlying trends [56]). The aim of this study was to investigate the impact of MRF addition on the microbial communities of the three main GI nutrient absorption sites (duodenum, jejunum, and ileum) of the *intestinum tenue* (“small intestine”) and the cecum in broilers. By removing intrinsic and extrinsic biases from 16S rRNA gene counts and cumulative community structures, we aim to highlight otherwise overlooked microbial taxa that may be of importance in food safety microbiology.

## Methods

### Sample collection and preservation

This broiler trial was performed at a commercial production site within the European Union. On the day of hatch, chicks were taken from a commercial hatchery and transported to an associated commercial farm. Approximately 35,000 birds were placed from the hatchery into each of two sheds where they received a control standard commercial wheat-soya diet or a standard diet plus MRF (Alltech Biotechnology) at the following inclusion rates; 1300:1000:600 g^t−1^ starter, grower, and finisher rations respectively. Birds were raised and fed as per typical commercial production conditions receiving feed and water ad libitum. All other conditions were kept uniform for both sheds. At day 35 (post-hatch) the intact gastrointestinal tracts of 12 randomly caught birds per shed were excised immediately after humane euthanisation. Intestinal contents from the duodenum, jejunum, ileum, and cecum were massaged into individual sterile tubes, immediately frozen on dry ice, transported within 8 h and stored at −80 °C for downstream processing.

### DNA extraction and 16S rRNA gene sequencing

DNA was extracted from intestinal contents using the QIAamp DNA Stool Mini Kit according to the manufacturer’s instructions using 0.05 g of intestinal content (QIAamp DNA Stool Mini Kit, Qiagen). Genomic DNA concentration was determined at a wavelength of 260 nm using a NanoDrop (NanoDrop). Isolated DNA was then used as a template in PCR amplification for construction of 16S rDNA libraries which were prepared and sequenced by BaseClear genomics. Sequencing libraries were prepared by amplification and barcoding of the 16S rRNA gene V3–V4 region and the resulting amplicons were sequenced on an Illumina MiSeq platform generating 10–50 k PE300 reads per sample. The mean library size used was 580 bp (inclusive of barcodes and adapters) and the insert size was approximately 460 bp (580–120 = 460 bp). A total 3,988,410 reads were achieved. In the control dataset, average reads for each of the duodenum, jejunum, ileum and cecum were observed to be 41,749.42 ± 6442.53, 45,074.75 ± 6468.97, 42,135.83 ± 7449.29, and 48,489.92 ± 4364.9 respectively. Comparatively, in the MRF-treated dataset average reads of 35,883.92 ± 4765.3, 35,644.5 ± 9590.25, 43,873.5 ± 6593.51, 39,495.67 ± 8224.7 for the duodenum, jejunum, ileum, and cecum were observed.

### Dataset construction

Each sample was adapter and quality trimmed using TrimGalore! *v.*0.6.6 [54]under default settings and powered with cutadapt *v.*3.0 [64] and FastQC *v.*0.11.9 [7]. Between 14,044 and 54,118 reads were observed pre-quality-control and between 13,868 and 53,830 after, with an observed percentage read discard range between 0.325% and 9.66%. Chimeras were identified using UCHIME *v.*4.2.40 [36] and removed*.* Quality controlled reads were merged using the “–fastq_merge” function in VSEARCH *v*.2.14.2 [37, 79 to give a single entry for each read pair in FASTA format.

### 16S rRNA database construction

A database of 16S rRNA genes was constructed by downloading all bacterial genome assemblies (*n* = 274,268) from NCBI assembly [51] and extracting all 16S rRNA genes using Barrnap *v.*0.9 (as used for rRNA detection by Prokka *v.*1.1.14 [83]) with default settings. Taxonomic lineages were assigned to each genome (and their associated genes) using the “lineage” function in TaxonKit *v.*0.6.0 [87] and standardised to the seven ranks (Domain, Phylum, Class, Order, Family, Genus, and Species) using the TaxonKit “reformat” function. Sequences with length less than 1200 nucleotides (nt) were discarded to mirror the strict filtering methods employed during the construction of the SILVA database [75]. Remaining sequences were searched against all other remaining sequences using the “–usearch-global” function in VSEARCH *v*.2.14.2 with a minimal percentage identity stringency score of 0.97 (97%), self-hits were excluded, and, with the exception of *Escherichia*, *Shigella*, and *Salmonella* (ESS), top-hit pairs where sequences were observed to be from different genera were discarded. The ESS species were excluded from further filtration during this step due to the close evolutionary relatedness of these clinically relevant genera [38, 43, 91]. Finally, exact duplicates of 16S rRNA genes were removed resulting in a database of 68,724 16S rRNA genes from 21,928 species from 37 definite phyla and 70 candidate phyla/divisions (107 in total). This dataset is available for download at (https://github.com/RobLeighBioinformatics/Broiler_GI_microbiome).

### Database weighting

Bacterial genomes are highly dynamic due to rapid gene duplication, loss, and horizontal transfer events which may result in varying numbers of 16S rRNA genes [95]. Alien and spurious 16S rRNA genes were removed during database construction, so it is anticipated that all genes in the database were chromosomal in origin. Species were weighted by the number of 16S rRNA genes remaining in each genome after the strict filtration steps during database construction. The median number of 16S rRNA genes was taken where multiple genomes from the same species were retained. Genera weighting was calculated by excluding all genomes not definitively identified to species level (*eg.* genomes labelled “*Salmonella* sp.” (as opposed to, for example, *Salmonella enterica* or “undefined Lactobacillaceae”) and assumed to be the median for all species in a given genus. For higher taxonomic ranks, the median of rank medians was taken (*eg.* for families, the median of all genera medians in each family was taken). This method was employed to prevent biasing from well sampled species in a genus compared to less common species (*eg. Escherichia coli vs. Escherichia marmotae*). This weighting table is available at https://github.com/RobLeighBioinformatics/Broiler_GI_microbiome.

### Taxonomic assignment and weighting

Each read entry was searched against our 16S rRNA database using the “–usearch-global” function in VSEARCH and top hits with an alignment stringency cut-off of 0.97 (97%) were extracted (Additional file 1: Tables S1–S6). To mitigate taxonomic misassignment, the stringency cut off was increased to 0.99 (99%) for species level assignment. Read counts were then weighted using the 16S rRNA gene counts calculated above (Additional file 1: Tables S7–S12). The proportion of each weighted taxon in each sample was computed and normalised (closed) by dividing by a “closure constant” (CC) for each sample and dividing each weighted read count per taxa by the closure constant (Additional file 1: Tables S13–S18). This standardisation ensures all samples have the same number of reads for downstream comparative analysis. The standardisation constant was constructed using the formula:

$${\text{CC}} = \frac{{\Sigma_{x} }}{{\max \left( {\Sigma_{{x_{1} }} , \Sigma_{{x_{2} }} , ..., \Sigma_{{x_{n} }} } \right)}}; \;{\text{CC}} \le 1$$

where *x*: Series of reads in a sample/replicate.

### Outlier processing

Due to the extensive intersample variation observed in microbiome studies [99], as discussed previously, we endeavoured to remove extreme outliers to examine potential underlying trends that may be otherwise obfuscated. Outliers were removed and imputed with the median of the remaining inliers using uniForest *v.*1 with default parameters [56]*.*

### Fold changes

For all comparisons made below, median fold changes (η_FC_) were calculated using the formula:

$$\eta_{{{\text{FC}}}} = \frac{{\eta_{(b)} - \eta_{(a)} }}{{\eta_{(a)} }}$$

where η_(*x*)_: Median observation for group *x.*

Fold changes have a lower limit of −1 (complete depletion) and no change is represented by 0. A FC is incalculable if η_(*a*)_ = 0 as this represents a complete introduction.

### Statistical analysis

Kolmogorov-Smironov tests [53, 92] using a Lilliefors’ distribution [59] were used to determine sample series distribution normality (H_0_:*X* ~ *N*(μ,σ^2^;H_A_:*X*≁*N*(μ,σ^2^); *P* > 0.05: *X* ~ *N*(μ,σ^2^)) and as all distributions were determined to follow a non-normal distribution, Brunner–Munzel tests [20] were used to compare taxa between the control and MRF treated datasets H_0_:*B* = 0.5;H_A_:*B* ≠ 0.5). A Brunner–Munzel test was used instead of a Mann–Whitney *U* test [62] as the data was assumed to have unequal variance due to the high level of variability usually observed in microbiome analyses [99]. A Bonferroni–Dunn (BD; *P*_BD_) correction [16, 35] was applied to each test (*P*_BD_ = *P* × *n*_comparisons_) and instances where *P*_BD_ ≤ 0.05 were considered to be statistically significant (Additional file 1: Table S19) and the FC (as described above) was used to indicate the trend changes. Different *n*_comparisons_ were used to calculate *P*_BD_ (by taxonomic rank) to strengthen confidence in results at lower taxonomic ranks, however, to restrict an overly stringent correction, statistical comparisons were only performed when η_Control_ or η_MRF_ > 20 (or η_site(*a*)_ or η_site(*b*)_ > 20).

### Ecological statistics

A bias-corrected Chao1 richness estimator [24], Simpson’s *D* index [90], Simpson’s *E* index [90], and Shannon’s *H* index [85] was calculated for each anatomical site in each dataset at each taxonomic rank using the sklearn-bio (skbio) *v.*0.2.0 Python library (http://scikit-bio.org/). A Brunner–Munzel test (H_0_:*B* = 0.5;H_A_:*B* ≠ 0.5) was performed between diversity indices at each rank. A Bonferroni–Dunn correction was performed for each subset (*n*_comparisons_ = 4) and instances where *P*_BD_ ≤ 0.05 were considered statistically significant (Additional file  1: Table S20). Statistical trend changes were determined using the FC calculation described above.

A principal component analysis [47, 73] (PCA) was performed between all data subsets at each site using the “PCA” module in the “sklearn.decomposition” Python machine learning library. A permutational analysis of variance [4] (PERMANOVA) was used to compare control *vs* MRF treated samples. A PERMANOVA is used to compare the centroid and dispersion of two groups based on the 2 dimensional (2D) or 3D coordinates of their points using 999 iterations (*i*_*n*_ = 999). A Bonferroni–Dunn correction was applied (*n*_comparisons_ = 4) and a *P*_BD_ ≤ 0.05 was considered statistically significant (Additional file 1^1^: Table S21).

A Bray–Curtis distance matrix [19] was constructed between control and MRF-treated datasets for each anatomical site using the “beta_diversity” driver function from the “skbio.diversity” Python library and a principal coordinate analysis (PCoA) was performed on each distance matrix using the “pcoa” function from the “skbio.stats.ordination” package. A PERMANOVA was used to compare control *vs* MRF treated PCoA groups using 999 iterations (*i*_*n*_ = 999) as is common practice. A Bonferroni–Dunn correction was applied (*n*_comparisons_ = 4) and a *P*_BD_ ≤ 0.05 was considered statistically significant (Additional file  1: Table S21).

### Short chain fatty acid concentration analysis

The concentrations of three short chain fatty acids (SFCA; acetate, propionate, and butyrate) in cecal digesta was measured using gas chromatography after metaphosphoric acid derivation as previously described with minor modifications [77]. Briefly, 0.20 g of thawed sample was diluted with 2 mL double-distilled water in a sterile screw-capped tube, then homogenized, and centrifuged at 4000 × g for 10 min at 10 °C. A volume of 1 mL of supernatant was then transferred to another Eppendorf tube and mixed with 0.2 mL, 25% (wt/vol) ice-cold metaphosphoric acid solution. Subsequently, this solution was kept at − 20 °C for 4 h. Samples were then thawed, 0.1 mL 4 M sodium hydroxide solution added and centrifuged at 4000 × g for 10 min at 10 °C before analysis. The supernatant was then filtered with a 0.22 μm membrane, and an injection volume of 0.4 μL of sample solution was analyzed using a gas chromatography (Agilent 7890A system) coupled with a CP-Wax 58 FFAP CB column (Agilent) and flame ionization detector to determine SCFA concentrations in cecal content. The concentrations of acetate, propionate, and butyrate were calculated and expressed as μmol/g of wet cecal digesta.

Again, Kolmogorov-Smironov tests (using a Lilliefors’ distribution) were used to determine sample series distribution normality (H_0_:*X* ~ *N*(μ,σ^2^);H_A_:*X*≁*N*(μ,σ^2^); *P* > 0.05: *X* ~ *N*(μ,σ^2^)) for control and MRF-treated SFCA concentration series. Equivarience was assessed using a Levene’s test (H_0_:σ^2^_*a*_ = σ^2^_*b*_;σ^2^_*a*_ ≠ σ^2^_*b*_) [57]. As equivariance was not observed between any pair and as one distribution (MRF-treated acetic acid) was determined to follow a non-Gaussian distribution, Brunner-Munzel tests were used to compare each taxon between the control and MRF treated datasets H_0_:*B* = 0.5;H_A_:*B* ≠ 0.5) (Additional file 1: Table S22).

## Results

### Broiler growth characteristics

The growth indices of the MRF supplemented broilers were compared with the control (Table 1). Feed conversion ratios and average live weights did not differ significantly between the two groups however, the MRF supplemented birds were on average 5 g heavier and finished 1 day earlier than the control group. Birds supplemented with MRF tended to have a greater European production efficiency factor (EPEF).

**Table 1:** Comparison of growth indices of broiler commercial units with and without MRF dietary supplementation

	Mean live weight (kg)	Age (d)	EPEF	FCR
Control	1.964	35.60	341.622	1.589
MRF	1.968	34.77	347.702	

### Effect of diet and GI tract section on α- and β- diversity

A total 3,988,410 sequence reads were recovered from the 96 samples analysed. In the control dataset, average reads for each of the duodenum, jejunum, ileum, and cecum were observed to be 41,749.42 ± 6442.53, 45,074.75 ± 6468.97, 42,135.83 ± 7449.29, and 48,489.92 ± 4364.9, respectively. Comparatively, in the MRF supplemented dataset average reads of 35,883.92 ± 4765.3, 35,644.5 ± 9590.25, 43,873.5 ± 6593.51, 39,495.67 ± 8224.7 for the duodenum, jejunum, ileum, and cecum, respectively.

**Fig. 1:**
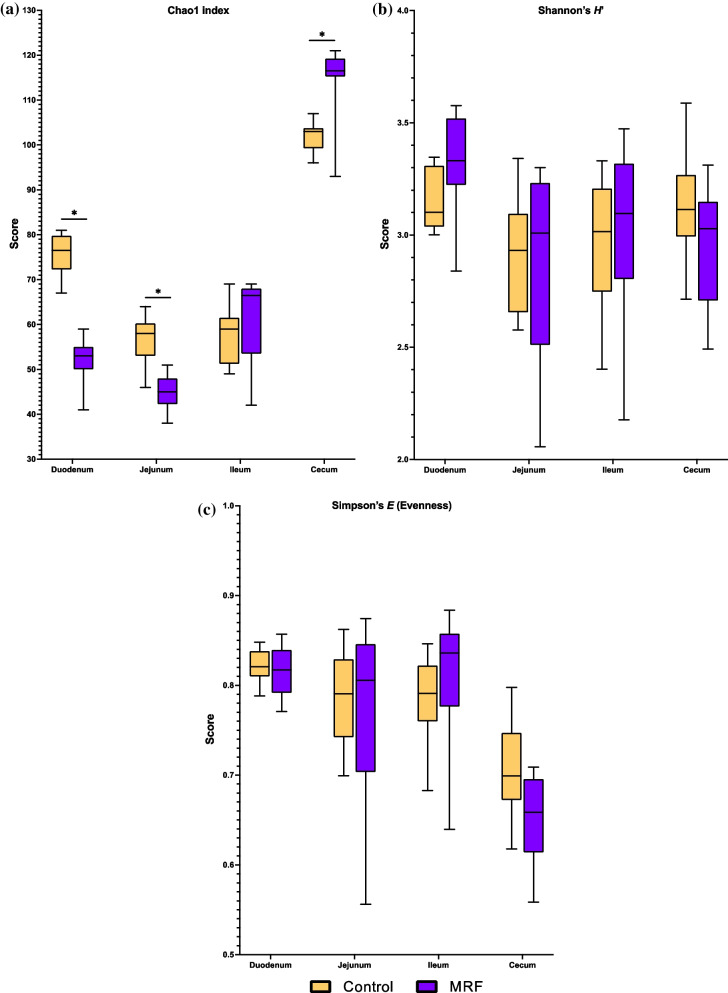
**a**–**c** Four α-diversity metrics displayed for the four anatomical sites explored in this study. Statistically significant (*P*_BD_ ≤ 0.05) results are highlighted with an asterisk

Microbial diversity at the four anatomical sites was estimated using α-diversity indices (Chao1 index, Simpson’s *E* (evenness), and Shannon’s *H’* index). Chao1 was used to estimate richness (Fig. 1a), Shannon's *H’* index was used to indicate diversity (Fig. 1(*b*..)) and Simpson’s *E* was used to indicate evenness (Fig. 1(*c.*); Additional file 1: Table S20). Richness was observed to be significantly increased in the MRF-treated ceca (Chao1:η_FC_ = 0.1311) and significantly lower in MRF-treated duodena (Chao1:η_FC_ = -0.3072) and jejuna (Chao1:η_FC_ = −0.2241) respectively. Evenness was not observed to be significantly affected by MRF-addition and the ileum was not observed to be modulated post-treatment.

**Fig. 2:**
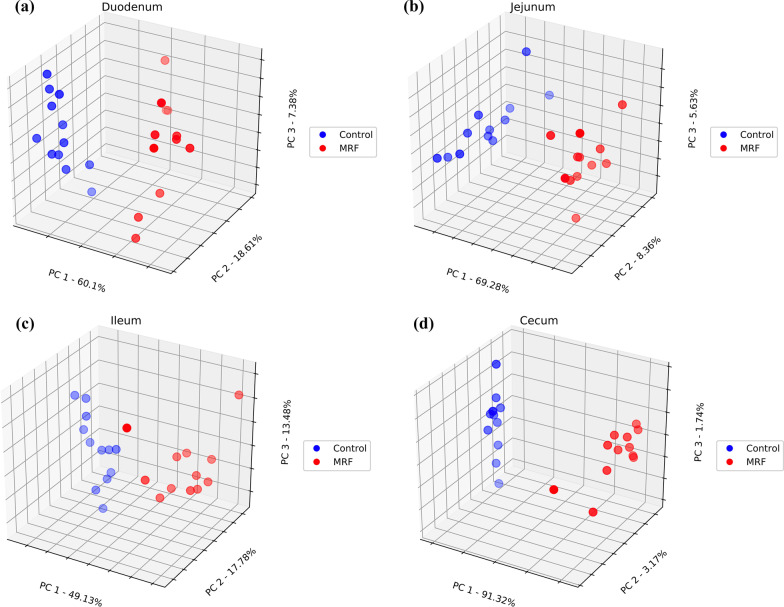
**a***–***d** Species-level Bray–Curtis distance matrices (β-diversity) expressed as PCoA between control (red) and MRF-supplemented broilers (blue) at each anatomical site

**Fig. 3:**
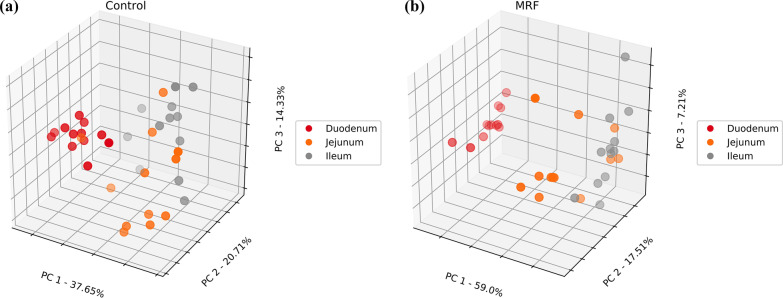
**a**, **b** Species-level Bray–Curtis distance matrices (β-diversity) expressed as PCoA between duodenal (red), jejunal (orange), and ileal (grey) anatomical sites across the control (left) and MRF-supplemented (right) datasets

Differences in β-diversity within the intestinal microbial population between groups and between intestinal sections within groups were assessed using PCoA (Figs. 2 and 3). The PCoA plots shown in Fig. 2a–d show that the bacterial community composition at the species level differed significantly (*P*_BD_ ≤ 0.05) as a result of diet in each intestinal section with PC1 accounting for 60.1%, 69.28%, 49.13% and 91.32% of the total variation; PC2 accounting for 18.61%, 8.36%, 17.78% and 3.17%; and PC3 accounting for 7.38%, 5.63%, 13.48%, and 1.74% in the duodenum, jejunum, ileum, and cecum respectively. The bacterial community composition between intestinal sections was also analysed for differences and showed that each intestinal section harboured a distinct bacterial community structure regardless of diet (Fig. 3a, b, *P*_BD_ ≤ 0.05).

### Effect of diet and GI tract section on bacterial community composition

To determine which bacterial taxa contributed to separating bacterial communities based on diet and intestinal section, the phylum level relative abundances of each GI tract were considered (Table 2). At the phylum level, four main bacterial phyla were identified within each gastrointestinal section, Actinobacteria, Bacteroidetes, Firmicutes, and Proteobacteria (newly renamed as Actinomycetota Bacteroidota, Bacillota, and Pseudomonadota, respectively [72]). Phylum Firmicutes was the predominantly abundant phylum within each GI section. Following MRF supplementation, Firmicutes were significantly lower in the duodenum, and significantly greater in the cecum. Actinobacteria was identified as the second most abundant phylum in all control group anatomical sites but was significantly lower in the duodenum and cecum as a result of MRF supplementation. Proteobacteria were significantly greater in the duodenum and significantly lower in the ileum following MRF addition to the diet. Finally, Bacteroidetes was predominantly detected in the cecum compared to any other site.

**Table 2:** Relative abundances of bacterial phyla obserbed in each anatomical site in both control and MRF supplemented broilers

	Duodenum			Jejunum			Ileum			Cecum		
	Control (η_%_)	MRF (η_%_)	FC	Control (η_%_)	MRF (η_%_)	FC	Control (η_%_)	MRF (η_%_)	FC	Control (η_%_)	MRF (η_%_)	FC
Firmicutes	**77.64**	**52.87**	−0.319^*^	87.11	96.66	0.110	88.49	87.81	−0.008	**74.76**	**91.17**	0.220^*^
Actinobacteria	**18.00**	**2.59**	−0.856^*^	12.12	2.84	−0.766	9.28	11.96	0.288	**13.89**	**1.88**	−0.864^*^
Proteobacteria	**4.08**	**44.45**	9.902^*^	0.81	0.36	−0.553	**2.55**	**0.23**	−0.908^*^	1.75	3.34	0.908
Bacteroidetes	0.02	0.04	N/A	0.00	0.00	N/A	0.00	0.00	N/A	9.57	3.99	−0.583

Significant differences (*P*_BD_ ≤ 0.05) are denoted by a superscript asterisk (*) and emboldened for each row in each intestinal section. Data associated with significance are also emboldened. Increases are denoted by positive fold changes whereas decreases are denoted by negative fold changes

The top 10 most abundant bacterial genera and species for each GI tract section in control and MRF supplemented groups are shown in Tables 3 and 4 respectively. At the genus level the most abundant genera within the *intestinum tenue* in both control and MRF supplemented groups were Lactobacillus followed by Bifidobacterium (> 90% abundance combined). In the MRF supplemented birds the duodenum samples were dominated by Proteobacterial genera *Pseudomonas*, *Halomonas*, and *Shewanella*. For the control dataset the most abundant species within the *intestinum tenue* were *Bifidobacterium animalis, Lactobacillus crispatus,* and *Lactobacillus salivarius* (accounting for a η_%_ > 65%). Comparatively, in the MRF-treated sample dataset, each *intestinum tenue* site had a distinct set of predominant species (*Bifidobacterium animalis, Lactobacillus aviarus, Lactobacillus crispatus,* and *Lactobacillus kitasatonis*; (however these were observed in highly divergent η_%_ between sites)) and alongside other species (listed below) accounted for η_%_ < 60% in all sites. For the duodenum, *Pseudomonas veronii,* and *Pseudomonas* sp. TKP were highly observed, and for the ileum, *Lactobacillus vaginalis* was also highly observed. In the cecum the most abundant genus was *Faecalibacterium* in both control and MRF supplemented groups (> 50%) followed by *Bifidobacterium* and *Blautia* in the control group and *Blautia* and *Lactobacillus* in the MRF supplemented group. For the control cecal dataset, the most abundant observed species were *Faecalibacterium* sp. An122, *Bifidobacterium gallinarum, and Bifidobacterium pullorum,* (accounting for a η_%_ > 65%). In the cecal MRF supplemented dataset, the most prominent species (accounting for η_%_ > 69%) were *Faecalibacterium* sp. An122*, Blautia* sp. An81*,* and *Eubacterium* sp. An11.

**Table 3:** The (ten) most prevalent bacterial genera observed at each anatomical site in both control and MRF-treated datasets

Site	Rank	Control (Genus)	η_%_	MRF (Genus)	η_%_
Duodenum	1	*Lactobacillus*	71.153	*Lactobacillus*	48.579
	2	*Bifidobacterium*	21.083	*Pseudomonas*	34.323
	3	*Pseudomonas*	3.670	*Halomonas*	7.813
	4	*Clostridioides*	2.305	*Shewanella*	4.285
	5	*Halomonas*	0.745	*Bifidobacterium*	2.223
	6	*Shewanella*	0.554	*Faecalibacterium*	0.673
	7	*Staphylococcus*	0.301	*Blautia*	0.651
	8	*Faecalibacterium*	0.238	*Staphylococcus*	0.234
	9	*Escherichia*	0.142	*Corynebacterium*	0.189
	10	*Blautia*	0.139	*Ruminococcus*	0.108
Jejunum	1	*Lactobacillus*	81.649	*Lactobacillus*	94.982
	2	*Bifidobacterium*	12.749	*Bifidobacterium*	3.564
	3	*Clostridioides*	1.785	*Staphylococcus*	0.218
	4	*Escherichia*	0.517	*Pseudomonas*	0.160
	5	*Agarivorans*	0.196	*Corynebacterium*	0.132
	6	*Pseudomonas*	0.146	*Faecalibacterium*	0.087
	7	*Halomonas*	0.045	*Blautia*	0.051
	8	*Streptococcus*	0.040	*Halomonas*	0.026
	9	*Staphylococcus*	0.036	*Streptococcus*	0.025
	10	*Shigella*	0.035	*Weissella*	0.022
Ileum	1	*Lactobacillus*	84.029	*Lactobacillus*	87.716
	2	*Bifidobacterium*	9.845	*Bifidobacterium*	9.994
	3	*Escherichia*	2.053	Ca*. Arthromitus*	1.015
	4	Ca*. Arthromitus*	1.501	*Corynebacterium*	0.173
	5	*Clostridioides*	0.443	*Escherichia*	0.094
	6	*Streptococcus*	0.429	*Staphylococcus*	0.082
	7	*Shigella*	0.258	*Romboutsia*	0.075
	8	*Agarivorans*	0.135	*Pseudomonas*	0.051
	9	*Romboutsia*	0.110	*Agarivorans*	0.021
	10	*Corynebacterium*	0.049	*Jeotgalicoccus*	0.017
Cecum	1	*Faecalibacterium*	51.748	*Faecalibacterium*	54.531
	2	*Bifidobacterium*	15.388	*Blautia*	11.548
	3	*Blautia*	5.372	*Lactobacillus*	7.422
	4	*Barnesiella*	3.716	*Eubacterium*	3.423
	5	*Lachnoclostridium*	3.588	*Lachnoclostridium*	3.232
	6	*Ruminococcus*	2.689	*Ruminococcus*	3.125
	7	*Eubacterium*	2.503	*Bacteroides*	2.364
	8	*Bacteroides*	2.364	*Bifidobacterium*	2.036
	9	*Lactobacillus*	2.117	*Pseudoflavonifractor*	1.823
	10	*Escherichia*	1.734	*Barnesiella*	1.675

Median relative abundances (η_%_) were used to determine the rank of each taxon in each subset

**Table 4:** The (ten) most prevalent bacterial species observed at each anatomical site in both control and MRF-treated datasets

Site	Rank	Control (Species)	η_%_	MRF (Species)	η_%_
Duodenum	1	*Lactobacillus crispatus*	26.636	*Pseudomonas veronii*	34.906
	2	*Bifidobacterium animalis*	21.394	*Pseudomonas* sp. TKP	14.440
	3	*Lactobacillus salivarius*	21.043	*Lactobacillus aviarius*	8.370
	4	*Lactobacillus reuteri*	7.874	*Lactobacillus kitasatonis*	6.143
	5	*Pseudomonas veronii*	2.766	*Shewanella algae*	6.010
	6	*Lactobacillus kitasatonis*	2.676	*Halomonas chromatireducens*	4.046
	7	*Clostridioides difficile*	2.447	*Bifidobacterium animalis*	3.062
	8	*Lactobacillus acidophilus*	2.386	*Lactobacillus crispatus*	2.621
	9	*Lactobacillus aviarius*	2.020	*Halomonas* sp. 1513	2.284
	10	*Lactobacillus agilis*	1.932	*Halomonas* sp. JCM 19,032	2.042
Jejunum	1	*Lactobacillus crispatus*	36.478	*Lactobacillus aviarius*	36.706
	2	*Bifidobacterium animalis*	16.176	*Bifidobacterium animalis*	13.624
	3	*Lactobacillus salivarius*	12.653	*Lactobacillus crispatus*	9.631
	4	*Lactobacillus aviarius*	5.401	*Lactobacillus reuteri*	8.691
	5	*Lactobacillus reuteri*	4.211	*Lactobacillus kitasatonis*	7.094
	6	*Lactobacillus kitasatonis*	3.877	*Lactobacillus acidophilus*	5.600
	7	*Lactobacillus acidophilus*	3.733	*Lactobacillus vaginalis*	4.961
	8	*Clostridioides difficile*	2.631	*Lactobacillus frumenti*	1.192
	9	*Lactobacillus johnsonii*	1.335	*Lactobacillus johnsonii*	0.815
	10	*Lactobacillus agilis*	1.227	*Lactobacillus pontis*	0.670
Ileum	1	*Lactobacillus crispatus*	36.279	*Bifidobacterium animalis*	23.394
	2	*Lactobacillus salivarius*	23.668	*Lactobacillus crispatus*	18.823
	3	*Bifidobacterium animalis*	13.321	*Lactobacillus kitasatonis*	17.042
	4	*Lactobacillus acidophilus*	3.406	*Lactobacillus aviarius*	8.292
	5	*Lactobacillus aviarius*	3.290	*Lactobacillus reuteri*	6.923
	6	*Escherichia coli*	2.986	*Lactobacillus vaginalis*	5.449
	7	*Lactobacillus reuteri*	2.938	*Lactobacillus acidophilus*	3.185
	8	*Lactobacillus kitasatonis*	2.446	*Lactobacillus johnsonii*	3.040
	9	Ca *Arthromitus* sp. SFB-rat-Yit	2.215	Ca*. Arthromitus* sp. SFB-rat-Yit	2.839
	10	*Lactobacillus agilis*	1.650	*Lactobacillus frumenti*	0.947
Cecum	1	*Faecalibacterium* sp. An122	53.414	*Faecalibacterium* sp. An122	57.393
	2	*Bifidobacterium gallinarum*	7.796	*Blautia* sp. An81	8.425
	3	*Bifidobacterium pullorum*	4.714	*Eubacterium* sp. An11	3.297
	4	*Barnesiella intestinihominis*	3.920	*Blautia hansenii*	2.432
	5	*Blautia* sp. An81	3.288	*Ruminococcus lactaris*	2.247
	6	*Lachnoclostridium* sp. An76	2.352	*Bacteroides fragilis*	2.213
	7	*Eubacterium* sp. An11	2.136	*Lactobacillus crispatus*	2.061
	8	*Bacteroides fragilis*	2.122	*Lachnoclostridium* sp. An76	2.045
	9	*Escherichia coli*	1.937	*Barnesiella intestinihominis*	1.784
	10	*Blautia hansenii*	1.925	*Pseudoflavonifractor* sp An184	1.385

Median relative abundances (η_%_) were used to determine the rank of each taxon in each subset

The relative abundances of several bacterial genera and species were significantly different with MRF supplementation (Tables 5 and 6, respectively). Notably, the bacterial genus *Escherichia* was significantly lower in the duodenum and ileum (numerically lower in jejunum and cecum, Additional file 1: Table S19). Genus *Shigella* was significantly lowered in the ileum, while the genus *Bifidobacterium* was significantly lowered in the duodenum and cecum. Whilst the genus *Lactobacillus* was noted to be significantly lower in the duodenum it was significantly greater in the cecum in MRF supplemented birds. Similarly, the genera *Anerostipes, Kineothrix*, and *Blautia* were noted to be significantly greater whilst *Alistipes* was significantly lower in the cecum of MRF supplemented birds when compared to the control. Genus *Clostridioides* was noted to be significantly lowered while other genera including *Shewanella*, *Pseudomonas,* and *Halomonas* were greater in the duodenum. Genera *Streptococcus* and *Agarivorans* were also significantly lower in the ileum of broilers supplemented with MRF. At the species level, the relative abundances of several bacteria were significantly different with MRF supplementation (Table 6). Of note, *Escherichia coli* and *Clostridoides difficile* were significantly lower across all three *intestinum tenue* sites following MRF supplementation. In the duodenum and jejunum, *Bifidobacterium gallinarum* was significantly lower, whereas *Bifidobacterium gallinarum* and *Bifidobacterium pullorum* were significantly lower in the cecum. Modulations in *Lactobacillus* species were observed throughout the GI tract following MRF supplementation. Of interest, *L. reuteri,* was observed to be significantly lower in the duodenum but significantly greater in the ileum and cecum and *L. salivarius*, was observed to be lower across the entire GI tract. The species *Barnesiella intestihominis* was noted to be significantly lower in the caeca of MRF-treated birds (compared to control birds), whereas *Blauta* sp. An81, which is strongly associated with weight gain, was observed to be significantly greater in both the cecum and jejunum. As mentioned above, *Escherichia coli* and *Clostridoides difficile* were observed to be significantly lower in the duodenum whereas *Pseudomonas veronii, Halomonas axialensis*, and *Shewanella algae* were significantly greater. After MRF-treatment, *Shigella flexneri* was observed to be significantly lower in the ileum.

**Table 5:** Significantly altered (increased or decreased) genera observed at each anatomical site

Site	Genus	η_Control_ (*n*)	η_MRF_ (*n*)	*P*_BD_	Change	FC
Duodenum	*Clostridioides*	936.993	0	0.0002	Decrease	Eradication
	*Escherichia*	57.812	5.770	0.0011	Decrease	−0.900
	*Bifidobacterium*	8571.620	903.799	1.49*e*^−08^	Decrease	−0.895
	*Lactobacillus*	28,928.722	19,750.855	5.43*e*^−08^	Decrease	−0.317
	*Shewanella*	225.335	1742.348	2.03*e*^−08^	Increase	6.732
	*Pseudomonas*	1492.102	13,954.595	9.60*e*^−09^	Increase	8.352
	*Halomonas*	302.917	3176.654	8.67*e*^−07^	Increase	9.487
Ileum	*Streptococcus*	174.335	4.979	0.0191	Decrease	−0.971
	*Escherichia*	834.585	38.234	0.0080	Decrease	−0.954
	*Shigella*	105.048	6.101	0.0007	Decrease	−0.942
	*Agarivorans*	54.887	8.581	0.0279	Decrease	−0.844
Cecum	*Alistipes*	116.939	2.955	0.0145	Decrease	−0.975
	*Bifidobacterium*	6256.469	827.671	0.0001	Decrease	−0.868
	Oscillospiraceae^[*is*]^	340.433	220.868	0.0009	Decrease	−0.351
	*Eubacterium*	1017.654	1391.518	0.0419	Increase	0.367
	Ruminococcaceae^[*is*]^	26.154	36.030	0.0451	Increase	0.378
	*Anaerostipes*	137.596	234.771	0.0125	Increase	0.706
	Firmicutes^[*is*]^	91.309	164.287	4.96*e*^−05^	Increase	0.799
	*Blautia*	2184.069	4695.135	3.97*e*^−05^	Increase	1.150
	*Kineothrix*	8.866	20.484	9.85*e*^−12^	Increase	1.310
	*Lactobacillus*	860.654	3017.700	0.0027	Increase	2.506

Standardised median read counts (*n*) are presented to illustrate the magnitude of the fold change.^[*is*]^ represents *incertae sedis* classifications

**Table 6:** Significantly altered (increased or decreased) species observed at each anatomical site

Site	Species	η_Control_ (*n*)	η_MRF_ (*n*)	*P*_BD_	Change	FC
Duodenum	*Lactobacillus salivarius*	21,043.166	773.255	0	Decrease	−0.9633
	*Lactobacillus crispatus*	26,636.067	2621.188	0	Decrease	−0.9016
	*Lactobacillus johnsonii*	1087.965	116.768	0	Decrease	−0.8927
	*Lactobacillus paragasseri*	153.621	19.226	0	Decrease	−0.8748
	*Bifidobacterium animalis*	21,393.561	3061.820	0	Decrease	−0.8569
	*Escherichia coli*	142.779	20.447	0.0045	Decrease	−0.8568
	*Lactobacillus helveticus*	37.155	6.869	0	Decrease	−0.8151
	*Bifidobacterium gallinarum*	84.895	16.604	8.04*e*^−09^	Decrease	−0.8044
	*Lactobacillus reuteri*	7873.725	1679.717	0	Decrease	−0.7867
	*Gardnerella vaginalis*	31.085	7.168	0	Decrease	−0.7694
	*Lactobacillus gallinarum*	186.486	50.006	7.70*e*^−15^	Decrease	−0.7318
	*Agarivorans* sp. Toyoura001	33.629	0	9.82*e*^−05^	Decrease	Eradication
	Ca*. Paraburkholderia calva*	15.977	0	0.0046	Decrease	Eradication
	*Chlamydia trachomatis*	3.859	0	0	Decrease	Eradication
	*Clostridia* sp UC5.1-1D1	22.984	0	0.0046	Decrease	Eradication
	*Clostridioides difficile*	2446.530	0	0	Decrease	Eradication
	*Intestinibacter bartlettii*	3.533	0	0.0449	Decrease	Eradication
	*Lactobacillus agilis*	1932.350	0	0	Decrease	Eradication
	*Lactobacillus hominis*	113.136	0	0	Decrease	Eradication
	*Lactobacillus psittaci*	23.226	0	0	Decrease	Eradication
	*Lactobacillus taiwanensis*	7.718	0	0	Decrease	Eradication
	*Lactobacillus ultunensis*	3.859	0	0	Decrease	Eradication
	*Pseudoflavonifractor* sp. An184	3.831	0	0.0449	Decrease	Eradication
	*Streptococcus macedonicus*	36.659	0	0.0216	Decrease	Eradication
	*Halomonas beimenensis*	3.859	13.737	0	Increase	2.5600
	*Pseudomonas marginalis*	7.803	43.309	0	Increase	4.5506
	*Shewanella chilikensis*	81.365	601.961	0	Increase	6.3982
	*Halomonas axialensis*	19.294	167.753	0	Increase	7.6944
	*Halomonas* sp. JCM 19,032	212.926	2042.185	0	Increase	8.5910
	*Halomonas meridiana*	174.590	1812.993	0	Increase	9.3843
	*Halomonas* sp. JCM 19,031	3.901	41.212	0	Increase	9.5639
	*Shewanella algae*	534.688	6009.597	0	Increase	10.2395
	*Pseudomonas* sp. KG01	11.339	130.232	0	Increase	10.4850
	*Pseudomonas veronii*	2765.991	34,906.046	0	Increase	11.6197
	*Pseudomonas* sp. TKP	1131.681	14,439.803	0	Increase	11.7596
	*Halomonas stevensii*	61.936	870.978	0	Increase	13.0625
	*Halomonas* sp. 1513	158.856	2283.586	0	Increase	13.3752
	*Pseudomonas* sp.	3.901	76.956	0	Increase	18.7259
	*Halomonas chromatireducens*	203.414	4045.677	0	Increase	18.8889
	*Halomonas boliviensis*	0	9.079	0	Increase	Introduction
Jejunum	*Clostridioides difficile*	2630.701	13.765	0	Decrease	−0.9948
	*Lactobacillus salivarius*	12,653.447	314.352	0	Decrease	−0.9752
	*Lactobacillus paragasseri*	928.751	69.853	0.0067	Decrease	−0.9248
	*Lactobacillus helveticus*	156.084	15.977	0	Decrease	−0.8976
	*Lactobacillus crispatus*	36,478.154	9630.854	3.35*e*^−14^	Decrease	−0.7360
	*Lactobacillus gallinarum*	390.921	206.510	0.0004	Decrease	−0.4717
	*Agarivorans* sp. Toyoura001	249.521	0	0.0003	Decrease	Eradication
	*Bifidobacterium gallinarum*	10.182	0	5.30*e*^−05^	Decrease	Eradication
	*Chlamydia trachomatis*	9.051	0	0	Decrease	Eradication
	*Curtobacterium* sp. PhB136	4.738	0	0	Decrease	Eradication
	*Intestinibacter bartlettii*	1.556	0	0.0046	Decrease	Eradication
	*Lactobacillus psittaci*	255.802	0	9.82*e*^−05^	Decrease	Eradication
	*Streptococcus macedonicus*	50.920	0	0	Decrease	Eradication
	*Lactobacillus hamsteri*	4.738	15.977	1.29*e*^−08^	Increase	2.3721
	*Lactobacillus oris*	120.946	547.410	0	Increase	3.5261
	*Lactobacillus vaginalis*	715.005	4961.468	1.23*e*^−10^	Increase	5.9391
	*Lactobacillus coleohominis*	17.771	152.119	0	Increase	7.5599
	*Lactobacillus frumenti*	44.772	1191.867	0	Increase	25.6207
	*Blautia* sp. An81	4.525	212.011	0	Increase	45.8504
	*Corynebacterium nuruki*	0	51.722	0.0225	Increase	Introduction
	*Ruminococcus lactaris*	0	22.171	0.0449	Increase	Introduction
Ileum	*Lactobacillus agilis*	1649.818	6.529	2.61*e*^−07^	Decrease	−0.9960
	*Lactobacillus salivarius*	23,668.295	214.878	0	Decrease	−0.9909
	*Clostridioides difficile*	571.044	6.340	0	Decrease	−0.9889
	*Streptococcus macedonicus*	593.510	7.163	0	Decrease	−0.9879
	*Escherichia coli*	2985.827	206.367	8.04e^−09^	Decrease	−0.9309
	*Shigella flexneri*	330.087	23.150	1.23*e*^−10^	Decrease	−0.9299
	*Lactobacillus helveticus*	232.948	54.550	3.06*e*^−10^	Decrease	−0.7658
	*Agarivorans* sp. Toyoura001	176.291	46.799	1.45*e*^−09^	Decrease	−0.7345
	*Lactobacillus hominis*	877.530	322.551	0.0131	Decrease	−0.6324
	*Lactobacillus paragasseri*	903.394	371.092	0.0013	Decrease	−0.5892
	*Lactobacillus hamsteri*	10.340	5.338	2.44*e*^−05^	Decrease	−0.4838
	*Bifidobacterium pullorum*	20.590	0	0.0449	Decrease	Eradication
	*Curtobacterium* sp. PhB136	5.170	0	0	Decrease	Eradication
	*Sanguibacter keddieii*	2.585	0	0	Decrease	Eradication
	*Shigella dysenteriae*	5.442	0	0.0449	Decrease	Eradication
	*Lactobacillus johnsonii*	1491.369	3040.351	1.23*e*^−10^	Increase	1.0386
	*Lactobacillus reuteri*	2938.173	6922.904	0	Increase	1.3562
	*Lactobacillus oris*	60.589	400.865	1.23*e*^−10^	Increase	5.6161
	*Lactobacillus kitasatonis*	2446.266	17,042.197	5.16*e*^−07^	Increase	5.9666
	*Lactobacillus coleohominis*	12.934	124.808	0	Increase	8.6492
	*Lactobacillus vaginalis*	344.435	5449.077	7.70*e*^−14^	Increase	14.8203
	*Corynebacterium* sp. J010B-136	5.442	90.274	2.23*e*^−07^	Increase	15.5880
	*Lactobacillus frumenti*	25.941	947.442	1.11*e*^−06^	Increase	35.5225
	*Blautia hansenii*	0	17.352	0	Increase	Introduction
	*Corynebacterium provencense*	0	6.529	0	Increase	Introduction
	*Corynebacterium variabile*	0	47.163	3.06*e*^−10^	Increase	Introduction
	*Halomonas chromatireducens*	0	17.328	0	Increase	Introduction
	*Lactobacillus secaliphilus*	0	5.784	0.0449	Increase	Introduction
	*Lactobacillus taiwanensis*	0	13.058	0	Increase	Introduction
	*Ruminococcus* sp. OM05-10BH	0	6.529	0	Increase	Introduction
	*Streptococcus equi*	0	18.831	0.0232	Increase	Introduction
Cecum	*Bifidobacterium gallinarum*	7795.660	583.668	0	Decrease	−0.9251
	*Lactobacillus salivarius*	383.137	33.494	4.24*e*^−06^	Decrease	−0.9126
	*Alistipes putredinis*	144.043	12.770	0	Decrease	−0.9113
	*Bifidobacterium pullorum*	4713.817	530.608	0	Decrease	−0.8874
	*Streptococcus macedonicus*	70.588	17.664	0.0068	Decrease	−0.7498
	*Ruminococcus* sp. N15.MGS-57	24.697	8.918	0.0112	Decrease	−0.6389
	*Barnesiella intestinihominis*	3919.930	1784.091	0.0006	Decrease	−0.5449
	*Shigella dysenteriae*	16.465	8.105	0	Decrease	−0.5077
	Ruminococcaceae sp. D16	16.911	8.640	9.70*e*^−05^	Decrease	−0.4891
	Lachnospiraceae sp. OF09-33XD	67.408	38.218	2.25*e*^−05^	Decrease	−0.4330
	Oscillospiraceae sp. VE202-24	962.770	580.671	7.70*e*^−15^	Decrease	−0.3969
	*Flavonifractor* sp. An100	25.766	16.747	0.0259	Decrease	−0.3500
	*Blautia* sp. aa 0143	41.155	28.604	5.41*e*^−05^	Decrease	−0.3050
	*Bifidobacterium saeculare*	79.899	0	0	Decrease	Eradication
	*Bilophila wadsworthia*	16.465	0	0	Decrease	Eradication
	*Clostridium* sp. M62/1	16.465	0	0	Decrease	Eradication
	*Gordonibacter urolithinfaciens*	8.232	0	0	Decrease	Eradication
	*Staphylococcus cohnii*	8.232	0	0	Decrease	Eradication
	*Streptococcus gallolyticus*	8.232	0	0	Decrease	Eradication
	*Eubacterium* sp. An11	2135.550	3297.052	1.64*e*^−07^	Increase	0.54388855
	*Eubacterium ramulus*	32.081	50.966	6.45*e*^−05^	Increase	0.5887
	*Anaerostipes* sp. 494a	364.093	595.732	0.0010	Increase	0.6362
	Ruminococcaceae sp. AM07-15	25.766	50.241	0.0072	Increase	0.9499
	Firmicutes sp AF16-15	133.334	276.899	0	Increase	1.0767
	*Kineothrix alysoides*	23.010	52.783	0	Increase	1.2939
	*Blautia* sp. An81	3288.129	8424.566	0	Increase	1.5621
	*Ruminococcus* sp. 1xD21-23	5.882	15.572	0	Increase	1.6472
	*Ruminococcus* sp. Zagget7	39.210	106.628	0	Increase	1.7194
	*Lactobacillus gallinarum*	20.581	66.358	2.61*e*^−07^	Increase	2.2243
	*Blautia* sp. KGMB01111	14.315	50.617	3.73*e*^−05^	Increase	2.5360
	*Acutalibacter* sp. 1XD8-33	9.421	33.494	1.23*e*^−10^	Increase	2.5552
	*Lactobacillus crispatus*	532.710	2060.556	0.0003	Increase	2.8681
	*Lactobacillus johnsonii*	20.581	96.311	0	Increase	3.6797
	*Lactobacillus reuteri*	80.809	506.430	0	Increase	5.2670
	*Anaerostipes hadrus*	8.589	54.266	0	Increase	5.3182
	*Lactobacillus vaginalis*	24.162	287.730	1.23*e*^−10^	Increase	10.9086
	*Anaerofustis stercorihominis*	0	16.210	0	Increase	Introduction
	*Bacteroides* sp. D22	0	23.989	0.0449	Increase	Introduction
	*Blautia hominis*	0	8.105	0	Increase	Introduction
	*Blautia obeum*	0	125.604	0	Increase	Introduction
	*Blautia* sp. An249	0	16.872	2.25*e*^−05^	Increase	Introduction
	*Clostridium* sp. AM29-11AC	0	76.449	0	Increase	Introduction
	*Clostridium* sp. OF09-36	0	19.109	0	Increase	Introduction
	Firmicutes bacterium AM29-6AC	0	8.105	0	Increase	Introduction
	Firmicutes bacterium AM41-5BH	0	15.572	0	Increase	Introduction
	*Lachnoclostridium* sp. SNUG30386	0	8.105	0	Increase	Introduction
	Lachnospiraceae bacterium OF09-6	0	8.105	0	Increase	Introduction
	*Lactobacillus coleohominis*	0	8.374	0.0449	Increase	Introduction
	*Lactobacillus frumenti*	0	7.786	0.0449	Increase	Introduction
	*Lactobacillus helveticus*	0	16.210	0.0068	Increase	Introduction
	*Lactobacillus oris*	0	109.678	0	Increase	Introduction
	*Lactobacillus paragasseri*	0	23.257	0	Increase	Introduction
	*Lactobacillus psittaci*	0	17.279	0	Increase	Introduction
	*Ruminococcus* sp. A254.MGS-108	0	5.403	0	Increase	Introduction
	*Ruminococcus* sp. AF17-22AC	0	22.142	0	Increase	Introduction

Standardised median read counts (*n*) are presented to illustrate the magnitude of the fold change

**Fig. 4:**
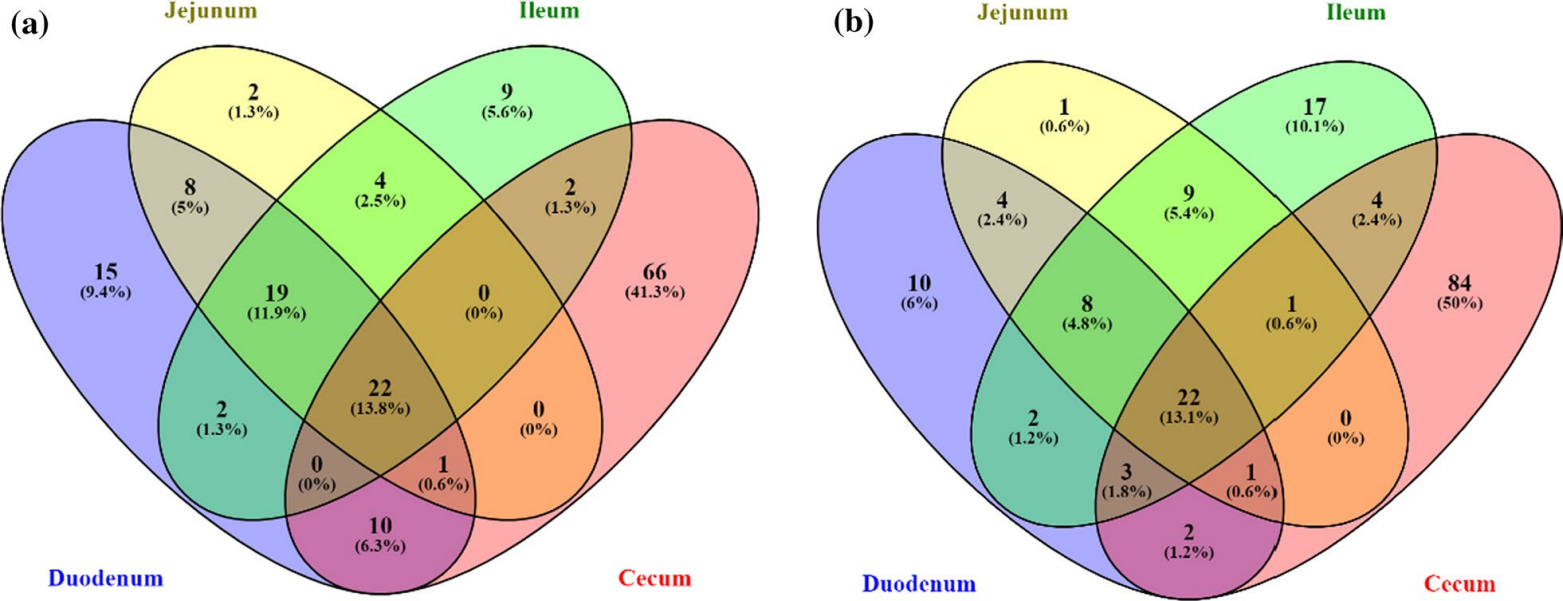
**a**, **b** Venn diagram showing common and shared species-level OTUs within each GI tract section for both control and MRF supplemented broilers (99% sequence identity)

To investigate the gut microbial community in different GI tract sections analysis of the common and unique OTUs was conducted, shown in the Venn diagrams (Fig. 4). A total of just 22 OTUs were shared by all 4 chicken gut sections in both the control and MRF supplemented groups. The number of OTUs observed in only one chicken gut section varied from 1 to 84, with the jejunum having the least amount of unique OTUs in both control (2) and MRF (1) supplemented groups and the cecum having the greatest amount of unique OTUs in both control (66) and MRF (84) supplemented groups. Neighbouring GI tract sections shared very few common OTUs with duodenum-jejunum sharing 8 and 4 OTUs, jejunum-ileum sharing 4 and 9 OTUs and ileum-cecum sharing 2 and 4 OTUs in control and MRF supplemented groups, respectively.

### Effect of diet on cecal short chain fatty acids

**Fig. 5:**
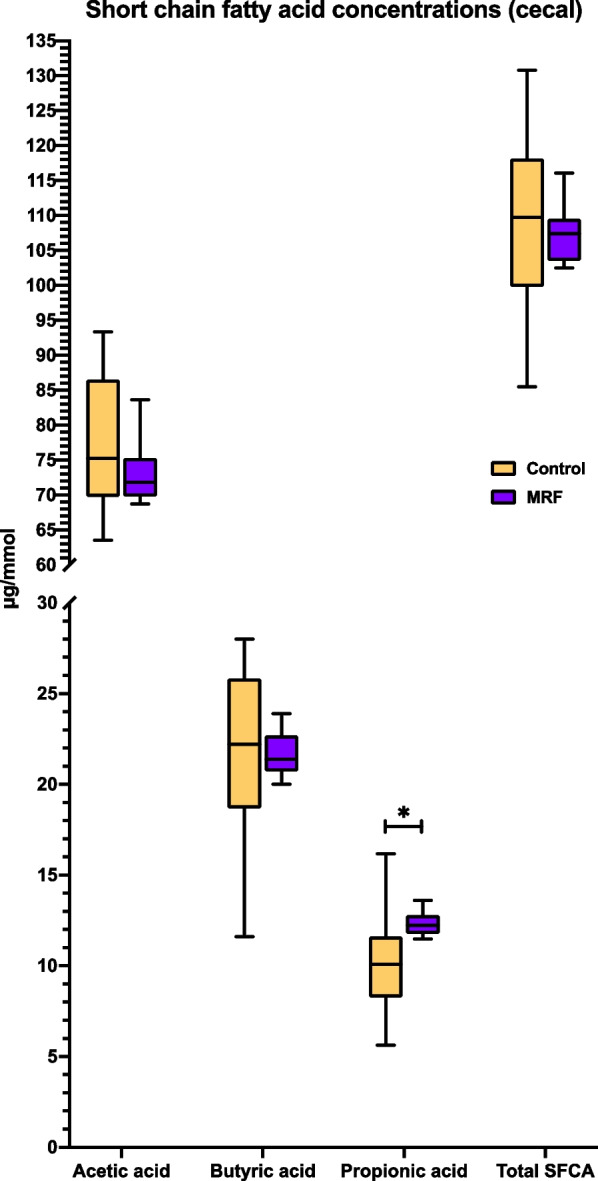
Short-chain fatty acid (SCFA) concentration in broiler ceca. Statistical significance is denoted using an asterisk

Cecal propionate was significantly greater (η_FC_ = 0.176) and cecal butyrate was numerically greater (η_FC_ = 0.009; *P*_BD_ = 1) in MRF supplemented birds when compared to the control (Fig. 5). No significant statistical differences in the concentrations of cecal acetate or total SCFA concentrations were observed between the control and MRF supplemented birds (*P*_BD_ > 0.05).

## Discussion

A large and diverse microbial community inhabits the broiler GI tract and contributes to overall health and growth efficiency by controlling pathogens, enhancing nutrient availability, and modulating immunological pathways (Borda-Molina, Seifert and Camarinha-Silva, 2018). Gastrointestinal microbiome composition and diversity is influenced by many external factors (*eg*. environment, age, breed, antibiotic use or dietary supplementation) which may yield beneficial or maleficial consequence [102]. In this study, the impact of MRF dietary supplementation on broiler GI tract microbiota (across the *intestinum tenue* and ceca) was explored. Supplemented birds were observed to finish one day earlier with higher average weight (5 g) and EPEF than their control counterparts (Table 1; indicating improved bird health and producer economic potential.

Bacterial species α-diversity indices of richness, diversity and evenness are scalable metrics of health status with higher diversity negatively correlated with dysbiosis [31, 52, 98]. Comparatively, β-diversity metrics are also measures of health, where low values are expected between samples and higher values are expected between treatment groups [26, 27]. Increased α-diversity and lower β-diversity in broilers can be achieved using pre- and probiotics, and such strategies positively correlate with improved FCR and feed efficiency [2, 46, 49, 94]. The results from this study agree with previous studies, whereby α- and β-diversity differ between anatomical site [25, 86, 101]. In particular, the cecum was observed to be most diverse, and the ileum to be least diverse of the four sites, and MRF impacted cecum α-diversity more than any *intestinum tenue* site (Fig. 1(*a*.-*c*.)). Despite the lack of intersectional *paries*, each section of the unidirectional *intestinum tenue* displays differential absorptive properties, yields dynamic environmental conditions (*e.g.* pH, water content, chemical profiles, and available O_2_ content [60]) and microbial compositional profiles [65]. As the *intestinum tenue* maintains a continual flow, perhaps it is not surprising that α-diversity is less impacted than the cecum which displays a cul-de-sac architecture.

Abiotic stressors or infection can reduce α-diversity, leading to dysbiosis [23, 45]; broiler cecal α-diversity reduction typically coincides with reductions in Lactobacillaceae and an increase in Enterobacteriaceae [21, 39]. While MRF supplementation effect on the *intestinum tenue* has not been explored prior to this study, the observed cecal results (highlighting the dysbiotic amelioration effect of MRF via community composition alteration and increases in α-diversity) are in agreement with previously published cecal studies [26, 27]. Additionally, diversity metric trends between control group anatomical sites are also in agreement with previously published results [42, 101].

The major bacterial phyla identified in each of the four GI tract sections included Firmicutes, Actinobacteria, Bacteroidetes, and Proteobacteria, with Firmicutes being most dominant throughout each section (Table 2). Bacteroidetes was lowly represented in the *intestinum tenue* and was found in most abundance in the cecum, mirroring observations in previous studies [18, 101]. The major bacterial genera across the *intestinum tenue* were *Lactobacillus* and *Bifidobacterium,* with *Lactobacillus* accounting for 48%-92% across these intestinal sections. Early studies [15, 34] also reported that the *intestinum tenue* microbiota was dominated by *Lactobacillus* and their conclusions have been independently confirmed using metagenomic analyses [18, 58]. Interestingly, the most abundant species within the *intestinum tenue* were distinct between control and MRF supplemented groups. *Bifidobacterium animalis, Lactobacillus crispatus,* and *Lactobacillus salivarius* dominated the control dataset throughout; comparatively, in the MRF-treated dataset, each *intestinum tenue* site had a distinct set of predominant species (Table 3). Through efficient carbohydrate fermentation, *Lactobacillus* are known to provide substantial aid to host metabolism, yielding improved feed conversion ratios and reduced mortality in broilers [76],*Lactobacillus* also deter pathogen adhesion to the lumen walls [61, 81]. Previous studies have shown that *Lactobacillus* can positively influence villus height (VH), crypt depth (CD) and VH:CD in broiler intestines [6, 58]. Increased VH and VH:CD are thought to provide a larger surface area and enhance ability of nutrient absorption [32].

Short-chain fatty acids (SCFAs) play an important role in gut physiology. Increased intestinal butyrate in broilers has been shown to have many positive effects including improved energy supply, intestinal villi development, microbiome modulation, anti-inflammatory properties, and enteric pathogen control [9]. In this study, the cecum was shown to be dominated by the bacterial families Ruminococcaceae, Lachnospiraceae, and Bifidobacteriaceae in the control group and Ruminococcaceae, Lachnospiraceae and Lactobacillaceae in the MRF supplemented group, with the genera *Faecalibacterium, Bifidobacterium, Blautia,* and *Lactobacillus* being most prominent. Cecal microbiota are generally dominated by strict anaerobes with many of these bacteria belonging to SCFA producing families Lachnospiraceae and Ruminococacceae [81]. The genus *Faecalibacterium* is a prominent butyrate producer and is correlated with enhanced epithelial health and reduced intestinal inflammation [69, 70, 100]. Prebiotic genera *Bifidobacterium, Blautia* and *Lactobacillus* also bioconvert complex carbohydrates to SFCA for host energy utilisation [14]. Increased SFCA concentration results in a lower gastrointestinal tract pH and de-conjugated bile acids, which aid in pathogen control [9, 63], 55]*.* While an insignificant butyrate increase (+ 0.95%) was observed post MRF-treatment, propionate (+ 21.41%) and SFCA producing *Blautia* were significantly increased in the cecum (+ 69%). These results corroborate previous suggestions that increased abundance of *Blautia* and *Faecalibacterium* abundances may be related to improved growth performance [103].

Potential foodborne pathogens *Escherichia coli* and *Clostridioides difficile* were significantly lower across the *intestinum tenue* and *Shigella flexneri* in the ileum. Mannan rich fraction binds type-1 fimbriae of Enterobacteraceae, and has been shown to lower the prevalence of these pathogens in the intestine of animals [1, 8, 41]. Reducing foodborne pathogens (from any source) promotes food chain integrity, with *Escherichia and Clostridioides* reported as being amongst the most concerning from a One Health perspective [82, 88]. Additionally, as these species are potentially toxicogenic, synthesised toxins may travel to distal sites of the host organism and remain in meat products postprocessing [5, 44, 68, 71]. As such, any reduction in their prevalence should be viewed as a positive outcome.

The probiotic *Bifidobacterium* spp. were also shown to be significantly lower in the jejunum, ileum, and cecum of MRF supplemented broilers and was noted previously in the broiler cecum [27]. An interesting result observed in this dataset was a significantly greater relative abundance of *Lactobacillus reuteri* in the ileum and cecum. When supplemented with *L. reuteri,* both mammalian and poultry models were observed to have considerably reduced Enterobacteriaceae, specifically *Salmonella enterica*, compared to non-supplemented controls [33, 97]. In addition to bacteriological protection, *L. reuteri* supplementation is observed to confer antiprotozoal activity against *Eimeria* spp. in turkeys [33] and against another Eimeriorinan (Apicomplexan) parasite, *Cryptosporidium parvum,* in immunodeficient mice [3]. In previous studies, *L. reuteri* was strongly associated with weight gain whereas *L. salivarius* was strongly associated with lean maintenance [33, 89, 97]. Interestingly, *L. reuteri* was increased and *L. salivarius* was decreased in MRF supplemented birds.

Dietary MRF supplementation was observed to yield significantly greater relative abundances of cecal bacterial genera from families Lachnospiraceae, Ruminococcaceae and Lactobacillaceae. Whilst these are typical of the main bacterial families found in the broiler cecum, modulating their abundances can have profound health impacts, such as reduced inflammation, reduced intestinal atrophy, and improved mucosal barrier function [66, 81]. The significantly higher relative abundances of probiotic genera *Lactobacillus* and *Blautia* in the cecum, alongside higher relative abundances of jejunal and ileal *Lactobacillus* indicate MRF prebiotic action [40]. In essence, the comprehensive impact of prebiotics have important host health benefits beyond that of simple microbiota modulation.

## Conclusion

This manuscript aimed to address the bird-to-bird (intersample) variation associated with microbiome studies and is the first to apply such corrections to a comparative supplementation study across intestinal geographies. Each GI tract section presented a distinct bacterial community composition which were altered as a result of MRF supplementation. Results from the present study indicated that *Lactobacillus* was the most abundant genus in the *intentinum tenue* and that the cecum was most bacterially divergent. Birds supplemented with MRF had significantly higher species richness in the cecum and significantly different bacterial community composition in each GI tract section. MRF supplemented birds had lower levels of the zoonotic pathogens *Escherichia, Clostridioides,* and *Shigella* which are of particular importance for food chain integrity. Higher levels of probiotic related bacteria, such as *Lactobacillus* and *Blautia*, were observed following MRF supplementation. Higher relative abundances of known SCFA producing bacteria (and SCFA concentrations) were also attributed to MRF supplementation. These bacterial and metabolite alterations highlight a protective role for dietary MRF inclusion to support broiler GI health and may allow safer meat to be produced.

## Supplementary Information

**Additional file 1**: Tables S1–S22.

## Data Availability

Data used for this study is available at https://github.com/RobLeighBioinformatics/Broiler_GI_microbiome. Sequence reads (fastQ files) will be deposited at NCBI SRA upon publication.

## Abbreviations

16S rRNA: 16 Svedbard ribosomal ribonucleic acid
2D/3D: 2 Dimensional/3 dimensional
ESS: *Escherichia–Salmonella–Shigella*
CC: Closure constant
IgA: Immunoglobulin A
*n*_*x*_: Number/count of *x*
PCA: Principal component analysis
PCoA: Principal coordinate analysis
PERMANOVA: Permutational analysis of variance
TR: Transformed reads (SI data)
TA: Relative abundance from transformed reads (SI data)
SI: Supplementary information
SFCA: Short chain fatty acid
Subsp.: Subspecies
*v.*: Version

## List of symbols

Δ: Difference
μ: Mean
σ: Standard deviation
σ^2^: Variance
η: Median
~: Approximal to
≁: Not approximal to
BD: Bonferroni–Dunn
FC: Fold change
H_0_: Null hypothesis
H_A_: Alternative hypothesis
*i*_*n*_: Number of iterations
*N*(μ,σ^2^): Normal (Gaussian) distribution
*P*: *P*-value
*P*_*BD*_: Bonferroni–Dunn corrected *P*-value
*X*: Sample distribution
